# What Does the Evaluation of the Organ Donation (Deemed Consent) Act 2019 in England Tell Us About the Effectiveness of Deemed Consent Systems for Deceased Organ Donation?

**DOI:** 10.1097/TP.0000000000005246

**Published:** 2024-10-23

**Authors:** Leah McLaughlin, Nicholas Mays

**Affiliations:** 1 School of Health Sciences, Bangor University, Bangor, United Kingdom.; 2 Policy Innovation and Evaluation Research Unit (PIRU), Department of Health Services Research and Policy, London School of Hygiene and Tropical Medicine, London, United Kingdom.

## Abstract

**Figure:**
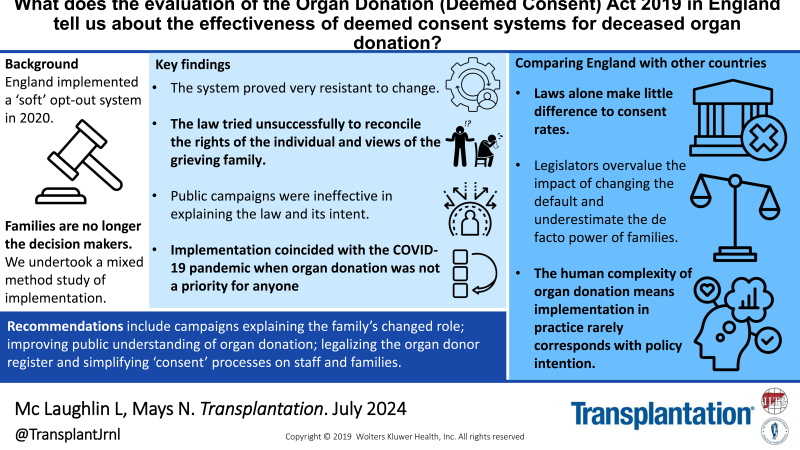

In the United Kingdom, as in most other countries, the demand for organs continues to exceed the supply, leading to many dying each year. England debated for some time whether to switch to an opt-out system of deceased donor consent as a way to address this important yet complex issue. Following Wales, which had introduced a “soft” opt-out in 2015, similar legislation was passed in England in 2019 and implemented in May 2020. The default switched to one that, in theory, supports deceased organ donation for those who meet specific criteria (often called deemed or presumed consent). Although the family remains essential to deceased organ donation by providing medical and biographical information to healthcare professionals to ensure the safety and effectiveness of the organs retrieved for transplant, according to the 2019 legislation, they are no longer the decision-makers. Instead, families are required to support the organ donation decisions their relatives make in life.

We undertook a mixed-method evaluation of the implementation of the law change comprising:

- a review of Parliamentary debates leading to the law change,
- a content analysis of the public’s responses to media articles ahead of the law change,
- analysis of intensive care and routine National Health Service Blood and Transplant potential donor audit data,
- surveys and interviews with relevant healthcare professionals,
- secondary analysis of public survey data and interviews with members of the public,
- interviews with relatives and close friends who had been approached about organ donation after their relative or friend had died, and
- a comparative analysis of England’s consent processes and documentation with Spain’s as a country with a higher consent rate.^1^

## KEY FINDINGS OF THE EVALUATION OF THE LAW CHANGE IN ENGLAND

**FIGURE 1.**
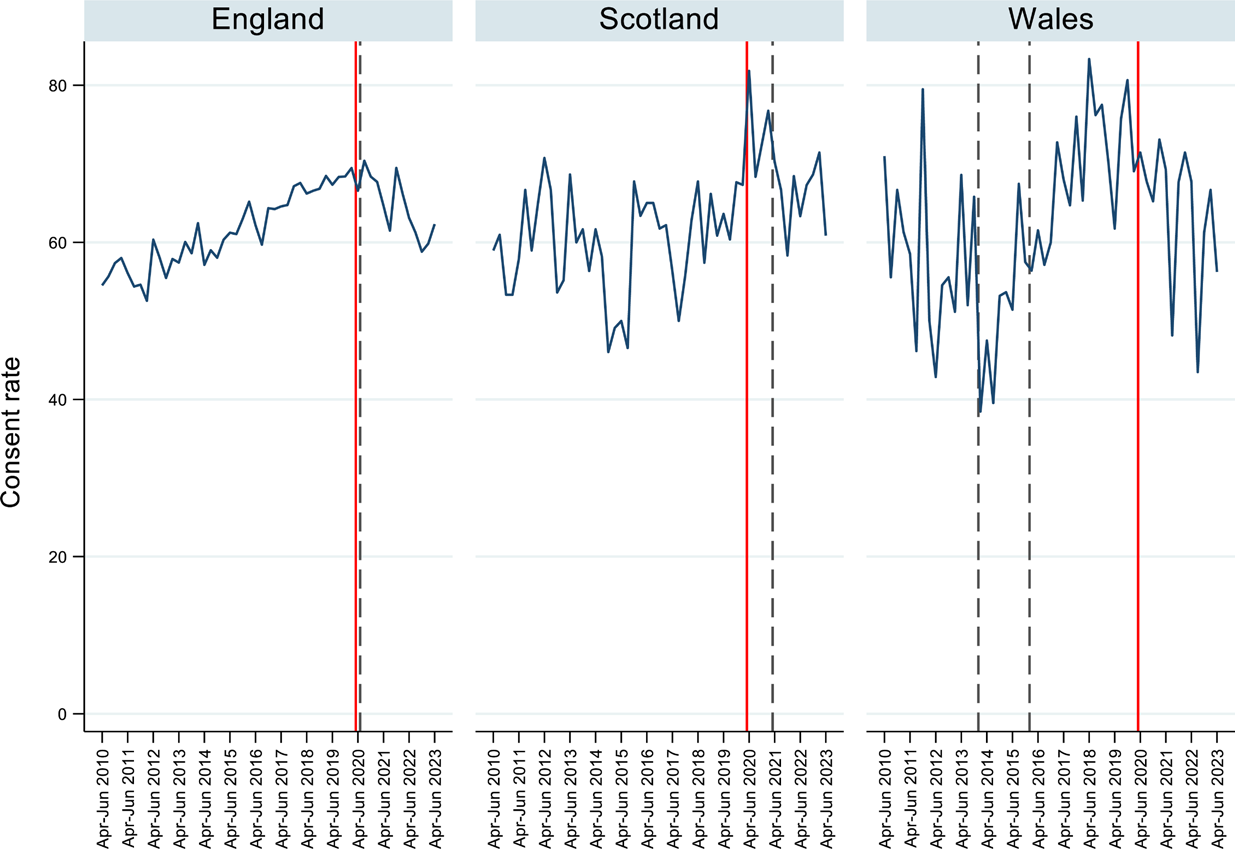
Trends in deceased organ donation consent rates before and after the opt-out law changes. Dashed lines indicate the implementation of the Act in practice. Wales first dashed line highlights the start of the implementation media campaign that accompanied the changes. Red lines indicate the start of the COVID-19 pandemic.

The principles behind the Act make it easier, in theory, for people to donate their organs and thereby save and improve the lives of others by giving decisions to individuals to make while they are alive. However, the ambitions of a “soft” opt-out have yet to be realized in either Wales or England. Consent rates have not yet increased despite similar opt-out laws (Figure 1).

This is for a number of reasons.

1. First, the system has proved very resistant to change. Deemed consent was added to the previous opt-in system. Family consent remains for some organs and existing consent forms and processes have been adapted (Table 1).
2. Second, the law was deliberately (very) “soft” to avoid any implication of an authoritarian state “taking” organs. It thus tries (unsuccessfully) to reconcile 2 competing goals—respecting the donation decision the potential organ donor made in life and respecting the right of family members to override that decision. The result is a confused system that tries to reconcile the 2 but satisfies neither.
3. Third, public campaigns were ineffective, deemed (or presumed) consent is (still) not understood by the public, and is far from being viewed as equal to a registered opt-in decision.
4. Fourth, the legislation was first implemented at the height of the pandemic when organ donation was not a priority for the public or the NHS.

**TABLE 1.** Hybrid system of deemed consent and opt-in to deceased organ donation in England

Organs and tissues included under the Act, that is, which deemed consent can lawfully be applied	Scenarios where deemed consent cannot be applied and first person consent is required, usually a family member			
Heart, transplanted either as a whole organ or for heart valves	All options are excluded if they are used for any form of **Advanced Therapy medicinal products**	All options are excluded if they are part of **a rare or novel transplant**	All options are excluded if they are part of any type of “**scheduled purpose.” This includes research, education, or training related to human health, clinical audit, quality assurance, and performance assessment**	**We often do not know at the time of consent what the organs and tissues will be used for.**
Lung(s)				
Liver, transplanted either as an organ or for liver cells (called hepatocytes)				
Kidneys				
Pancreas, transplanted either as a whole organ or for pancreatic cells that produce insulin (called islets)				
Intestinal organs (small bowel, stomach, abdominal wall, colon, spleen)				
Eye				
Nervous tissue				
Arteries/veins/blood vessels				
Bone				
Muscle				
Tendon				
Skin				
Rectus fascia (tissue that encases abdominal muscles)				

Organs and tissues excluded from the Act				
Arm, brain, face, finger, foot, forearm, hand, leg, lower leg, mouth, nose, spinal cord, thigh, toe, trachea, upper arm, cervix, clitoris, embryo, fallopian tube, fetus, labia, ovary, penis, perineum, placenta, prostate, testicle, umbilical cord, uterus, vagina, vulva	Will require another type of consent, that is, from the person before they died or first person, usually from a family member			

The Human Tissue (Permitted Material: Exceptions) (England) Regulations 2020, https://www.legislation.gov.uk/uksi/2020/521/made.

Organs and tissues to be excludedfrom the new system of organ andtissue donation in England (knownas “opt-out” or “deemed consent”), https://assets.publishing.service.gov.uk/media/5cc8101640f0b676837c2c5b/Organs_and_tissues_to_be_excluded_from_the_new_system_of_organ_and_tissue_donation_in_England_-_consultation_document.pdf.

## HOW THE CURRENT FINDINGS RELATE TO THE EXPERIENCE OF OTHER COUNTRIES

Changing the way that people are able to signal their decisions in relation to deceased organ donation has become somewhat of a global trend in recent years. On the face of it, the evidence in favor of shifting to some form of opt-out system would appear clear: of the top 10 countries with the highest number of deceased organ donations in 2022, 8 had opt-out systems.^2^ Nineteen of 27 European Union Member States currently implement opt-out organ donation systems, including those with the highest rates of donation. Taking one example, Iceland implemented an opt-out system in 2018 and subsequently jumped from mid/low in the global rankings to having some of the highest rates of donation in the world by 2022.

These findings suggest that opt-out is superior to opt-in. However, on closer investigation, the situation is far from straightforward. Opt-out systems are usually implemented and supported by other changes. Unpicking what difference legislation makes in isolation from associated system changes and preexisting factors such as health care resources (eg, numbers of specialist staff, intensive care unit beds, etc), culture, media coverage, trust in health and government agencies, end-of-life care practices, and population characteristics, such as general health and ethnic composition, remains challenging. Recent evidence shows that legislation alone makes little or no discernible long-term difference in making more organs available for transplant.^3^

Research indicates that the assumptions underpinning opt-out legislation should be treated with caution. Legislators, in particular, seem fairly consistently to overvalue the impact that a change in default that opt-out makes while underestimating the de facto role (and power) given to the family in a deceased organ donation context to override actual or deemed deceased donor decisions.^4^

Analyses of ways of increasing the rate of organ donation across the globe suggest that the focus needs to be on bolstering the existing healthcare infrastructure. What matters is the capacity and readiness of the healthcare system to support deceased donations rather than the precise terms of any particular legislation.^3,5^

Given the trend towards opt-out systems, a recent international consensus forum looked more closely at the relationship between the opt-out system and the mechanisms available to enable individuals to make their organ donation decisions known, particularly via organ donation registers. Registers appear to be even more variable in design and operation than opt-out systems. The forum concluded that registries need to be reformed to ensure that donation decisions made in life are as clear, up-to-date, and legally binding as possible, irrespective of whether the system is opt-in or opt-out.^6^ More research is needed to identify which forms of register best fit which type of healthcare system, especially in the context of an intention to change consent policy.

A recent comparison between England and the Netherlands is especially illuminating since each country is aiming to do exactly the same thing, but in very different ways: a mandated choice policy in the Netherlands in which all adults older than 18 y must make a decision in advance, by law, versus a default choice policy in England in which people are presumed to support being a deceased donor unless they say otherwise. The authors concluded it was too soon definitively to identify the impacts of either system, but the comparison highlights the variability in terms of how opt-out systems manifest in practice and reinforces the point that neither policy is likely to work in isolation from other health system supports.^7^

Research into the translation of organ donation policies into approaches made to families to discuss donation across the European Union found that pathway(s) to organ donation are underpinned by a highly varied mix of laws, regulations, guidelines, or combinations thereof, or even, nothing at all. The authors argue that practice rarely conforms to the content of formal policies due to the make-up of societies, cultural factors such as traditions, beliefs and values, and the context of acute bereavement.^8^

Finally, our own analysis of the documents and processes in the Spanish opt-out system (the world leaders in deceased organ donation) compared with the United Kingdom reinforces the need to focus beyond the specifics of legislation to look at wider societal features, the most striking being the higher priority given to organ donation in Spain.^9^

## IMPLICATIONS AND RECOMMENDATIONS FOR POLICY AND PRACTICE

Based on the evaluation of deemed consent in England, we have identified recommendations for changes that we think will narrow the gap between the goals of the opt-out legislation and the reality of its implementation.

The main recommendations are:

- To introduce new and ongoing public media campaigns. Communications need to emphasize the changed role of the family as well as improve public understanding of the circumstances likely to bring about deceased organ donation and the processes involved. Over time, the goal would be to institute a more positive philosophy of deceased organ donation in the general population, thereby enabling organ donation to be better embedded in end-of-life care.
- To give decisions on the organ donor register greater legal status to further legitimize and protect individuals’ decisions in life and increase support for the changed role of the family. There also needs to be regular reminders of decisions embedded in day-to-day life so that decisions are kept up-to-date, thereby helping the specialist nurses in organ donation in their roles.
- To shorten and simplify the documents and processes involved in deceased donation so that they only cover the essentials needed to ensure the safety and effectiveness of transplanted organs.
- To provide more staff training to implement deemed consent rather than the previous model of explicit consent to organ donation so that practice is more closely aligned with the intent of the opt-out legislation.
- To clarify the concept of deemed consent and increase public understanding of the principle so that family members consider it as a legitimate pathway to donation for their deceased relative.
